# Travel nurse work experiences: A comparison of staff and travel nurses' burnout and job attitudes

**DOI:** 10.1097/nmg.0000000000000123

**Published:** 2024-04-29

**Authors:** Paul E. Spector, Shani Pindek, Melisa R. Hayman, David J. Howard, Maryana L. Arvan

**Affiliations:** **Paul E. Spector** is an organizational behavior science contractor at the Florida Health Sciences Center-Tampa General Hospital and a part-time professor at the Muma College of Business, University of South Florida in Tampa, Fla. **Shani Pindek** is an assistant professor at the University of Haifa in Haifa, Israel. **Melisa R. Hayman** is the director of patient care services at the Muma Children's Hospital, Tampa General Hospital in Tampa, Fla. **David J. Howard** is the director of the People Development Institute at the Florida Health Sciences Center-Tampa General Hospital in Tampa, Fla. **Maryana L. Arvan** is a courtesy professor at the University of South Florida in Tampa, Fla.

## Abstract

A survey of 330 hospital RNs assessed burnout, job satisfaction, turnover intentions, and perceptions of work assignments. Understanding how travel nurses' work experiences differ from the experiences of staff nurses can help nurse leaders determine the best approaches to manage and support these nurses.

**Figure:**
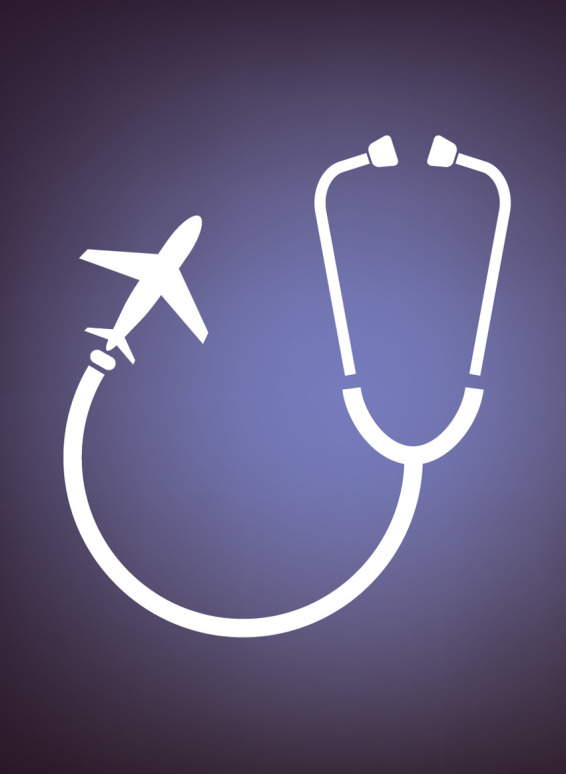
No caption available.

Hiring temporary travel nurses to address understaffing has been a strategy used by hospitals for some time, reaching a peak during the onset of the COVID-19 pandemic. With the widespread use of travel nurses, it's remarkable that so little research has been published concerning their work experiences. There are some research reports that focus on administrative issues such as costs or the impact on patient care.^1^,^2^ However, few studies have explored the travel nurses' experiences and whether their feelings about work differ from those of staff nurses.

An understanding of how travel nurses experience work and how their experiences might differ from those of staff nurses can inform the best approaches to manage and support travel nurses, which is vital for maintaining their well-being and ability to provide high-quality patient care. This article reports on a statewide survey of staff and travel nurses working in patient care in hospital settings, assessing their burnout, job satisfaction, turnover intentions, and perceptions of work assignments.

## Literature review

Nursing is an occupation with high levels of burnout.^3^ Burnout has been linked to patient safety, as well as job dissatisfaction and turnover, which have contributed to nursing shortages.^4^-^6^ Healthcare leaders have employed travel nurses to fill gaps related to understaffing.^7^ Although there are many published studies about nurses' experiences of working in hospital settings, few have been published specifically about travel nurses.

Faller and colleagues found that travel nurses had higher levels of burnout compared with another published study of employees in other occupations who were assessed using the same instrument.^8^ Similarly, Raso and Fitzpatrick surveyed travel nurses who reported an elevated or “at risk” level of emotional distress on the job, and they noted that levels were similar to a sample of staff nurses, although no statistical comparisons were provided.^9^ In a qualitative interview study, Ronnie identified issues of unfair task assignments reported by travel nurses.^10^ What they describe sounds much like the concept of illegitimate tasks from the general job stress literature.

Illegitimate tasks are defined as assignments that employees feel are inappropriate, either because someone else should be doing them (for example, it's not their job) or they shouldn't be done at all (for example, bureaucratic tasks perceived to be unnecessary).^11^ These studies identified potential sources of travel nurse dissatisfaction and job stress but provided no context because they didn't compare travel nurses with staff nurses. Such context is important to identify areas in which management needs to pay particular attention to travel nurses on their units.

## The current study

The current study used a quantitative survey design to compare the experiences of staff and travel nurses and included measures of burnout, job satisfaction, and turnover intentions, which are all concerns that have been noted for some time. A measure of pay satisfaction was also included, which the research team expected to be higher in travel nurses due to their substantially higher salaries; high pay is a major motivator for nurses to pursue travel contracts.^12^ As noted, Ronnie found that travel nurses felt that they were assigned mundane tasks in an unfair manner.^10^ Because the depiction of mundane tasks overlaps with the concept of illegitimate tasks, the researchers included a measure for illegitimate tasks. Finally, the researchers evaluated perceived workload. Given that travel nurses might perceive unfairness in work assignments, the researchers wondered if the travel nurses would perceive heavier workloads.

The study aimed to answer the following question: Do staff and travel nurses differ on burnout, job satisfaction, turnover intentions, and perceptions of work assignments?

## Methods

### Sample

The researchers surveyed RNs in Florida using a publicly available email list downloaded from the Florida Department of Health website. The design was cross-sectional (all data were collected in a single survey), which is commonly used in survey studies of nurses. Using the SurveyMonkey platform, researchers sent 87,008 invitations to nurses who were working in hospital settings and engaged in direct patient care asking them to complete the survey; 1,841 emails were returned as undeliverable, and 85,167 were successfully delivered to an inbox. The invitation noted that nurses had to work in direct patient care in a hospital setting to be eligible to participate. Of those emails delivered, 31.6% (26,878) were opened, according to SurveyMonkey analytics.

The landing page of the survey reiterated that participants had to be hospital nurses with direct patient-care responsibilities. After the informed consent page, the first question asked if the person worked in a hospital and provided direct patient care. Those who chose the “no” response were taken to the end of the survey. Of those who opened the email, 3.2% (864) clicked the survey link, and 373 provided at least partial data. Based on participants' responses to a question asking if the participant was a staff nurse or a travel nurse, the final sample included 29.8% (n = 111) travel nurses and 70.2% (n = 262) staff nurses.

### Measures

All variables were assessed with established measures. Coefficient alphas for the current study can be seen in the main diagonal of Table 3 for the combined sample (staff and travel nurses). Two components of burnout were measured with two of the three subscales from the 14-item version of the Shirom and Melamed measure.^13^ Physical fatigue (for example, feeling physically drained) consisted of five items, and emotional exhaustion (for example, having trouble concentrating) consisted of three items. The measures used a 7-point Likert-type scale ranging from “never or almost never” to “always or almost always.” This scale has been used in nearly 700 studies and has demonstrated high construct validity, factorial validity demonstrated in a confirmatory factor analysis, and predictive validity in relating to theoretically expected variables.^14^-^16^ Internal consistencies reported exceed the recommended minimum of .70 (.90 for physical fatigue and .82 for emotional exhaustion).^17^,^18^

Overall job satisfaction was measured with the job satisfaction subscale from the Michigan Organizational Assessment Questionnaire.^19^ It consists of three items and was evaluated with a 7-point Likert-type scale ranging from “disagree very much” to “agree very much.” Bowling and Hammond reported a mean internal consistency for the scale across 79 studies of .85, and significant predictive validities with many theoretically expected variables.^20^ The pay satisfaction subscale from the Job Satisfaction Survey was included.^21^ It had four items and used the same response choices as the overall job satisfaction scale. Spector reported a coefficient alpha for the scale of .75 and an 18-month test-retest reliability of .45. It showed a convergent validity coefficient of .66 with the popular Job Descriptive Index, and a significant correlation with salary level.^21^

Workload was assessed with the Quantitative Workload Inventory, a five-item measure using a 5-point summated rating scale, ranging from “less than once per month or never” to “several times per day.”^22^ Coefficient alpha was found to average .82 across 15 studies, and the workload scale significantly relates to both physical and psychological strains.^22^ Illegitimate tasks were measured with Matthews and colleagues' two-item measure, using the same response choices as the two job satisfaction measures.^23^ They report evidence for construct validity provided by confirmatory factor analysis and predictive validity based on significant correlations with expected variables. Turnover intention was assessed with a single item that asked how often the person seriously considered quitting, using a 6-point scale ranging from “never” to “extremely often.”^24^ This single-item scale relates as expected to job satisfaction and employee turnover.^25^

Four demographic items were included: nurses' main department, their highest level of education (from associate to doctoral degree), whether or not they supervised anyone (yes/no), and how many years they had been a nurse (see Table 1). The eight most frequently mentioned departments are shown in the table.

**Table 1:** Comparison of background variables for staff and travel nurses

	Staff nurse	Travel nurse	Statistical test
**Hospital department, n (%)**			
Acute care/medicine	91 (40.3%)	44 (43.1%)	Χ_2_(7) = 5.9, *P* < .5531
ICU	47 (20.8%)	23 (22.6%)	
OR	25 (11.1%)	15 (14.7%)	
Pediatric/neonatal	17 (7.5%)	3 (2.9%)	
Postoperative care	11 (4.9%)	7 (6.9%)	
ED	18 (8.0%)	6 (5.9%)	
Labor and delivery	11 (4.9%)	3 (2.9%)	
Medical/surgical	6 (2.7%)	1 (1.0%)	
**Education, n (%)**			
Associate degree	53 (20.2%)	29 (26.1%)	Χ_2_(3) = 2.0, *P* < .5743
Bachelor's degree	163 (62.2%)	62 (55.9%)	
Master's degree	41 (15.7%)	17 (15.3%)	
Doctoral degree	5 (1.9%)	3 (2.7%)	
**Supervisory role, n (%)**			
Yes	58 (22.1%)	3 (2.7%)	**X_2_(1) = 21.3, *P* < .0001**
No	204 (77.9%)	107 (97.3%)	
**Mean (SD) years as a nurse**	17.0 (12.2)	15.0 (10.9)	F(1, 369) = 2.1, *P* = .14

Note: Bold indicates a statistically significant difference

### Ethical considerations

The study protocol received an exempt approval from the University of South Florida institutional review board (IRB). The email invitation had basic informed consent information including that the survey was anonymous and voluntary. The landing page for the survey contained the detailed informed consent. As required by the IRB, the individual had to click that they agreed to participate to continue to the survey.

## Results

The researchers began the analysis by comparing the samples of staff and travel nurses to see if they differed in demographics. There were no significant differences between the two categories of nurses in hospital department, education level, or tenure as a nurse (see Table 1). There was a significant difference in supervisor status: 22.1% (n = 58) of staff nurses indicated they had supervisor responsibilities versus 2.7% (n = 3) of travel nurses (Χ^2^(1) = 21.3, *P* < .0001).

The researchers next conducted a series of analyses of variance (ANOVA) to compare the mean levels of the study's main variables for staff nurses and travel nurses. As shown in Table 2, travel nurses scored significantly higher on turnover intentions (*P* < .0123), the physical fatigue component of burnout (*P* < .0257), and illegitimate tasks (*P* < .0169). The differences between the two groups were statistically nonsignificant for overall job satisfaction (*P* < .1980), pay satisfaction (*P* < .9444), the emotional exhaustion component of burnout (*P* < .3966), and workload (*P* < .1459). Despite substantially higher pay for travel nurses, the pay satisfaction means for the two groups were almost identical.

**Table 2:** Comparison of staff and travel nurses on burnout, job attitudes, and work experiences

Variable	Staff nurse Mean (SD)	Travel nurse Mean (SD)	F	R^2^
Turnover intention	**3.6 (1.6)**	**4.0 (1.7)**	6.32∗	.02
Job satisfaction	14.5 (5.1)	13.8 (4.7)	1.66	.00
Pay satisfaction	11.2 (6.2)	11.1 (5.7)	0.00	.00
Emotional exhaustion	13.4 (4.7)	13.9 (5.1)	0.72	.00
Physical fatigue	**21.9 (7.7)**	**23.7 (6.7)**	5.02∗	.01
Workload	21.1 (4.3)	21.8 (4.2)	2.12	.01
Illegitimate tasks	**10.0 (3.0)**	**10.9 (3.0)**	5.76∗	.02

Note: Significant mean differences are bolded. Degrees of freedom for ANOVA was 1,371 for all but workload (1,344). Measures included: Michigan Organizational Assessment Questionnaire (job satisfaction), Job Satisfaction Survey (pay satisfaction), Shirom-Melamed Burnout Measure (emotional and physical fatigue), Quantitative Workload Inventory (workload), and Matthews and colleagues' (2022) single-item measures (illegitimate tasks).^13^,^19^,^21^-^23^

∗ *P* < .05

One possibility for the lack of differences between the two groups was that the staff nurses were more likely to be supervisors. The researchers controlled for supervisor status by repeating the analyses without the supervisors in the sample. Results were only trivially different, with the significant comparisons remaining significant at *P* < .05 and the nonsignificant comparisons remaining nonsignificant at *P* > .05.

Table 3 contains correlations among all the variables in the study for the two groups of nurses separately. For the staff nurses, all main variables in the study were significantly correlated at *P* < .05. This wasn't the case for the travel nurses, as emotional exhaustion wasn't significantly correlated with either workload or illegitimate tasks, and job satisfaction wasn't significantly correlated with illegitimate tasks. These results suggest that there might be some differences between staff and travel nurses in relationships among some of the variables included in the study.

**Table 3:** Correlations among nurse burnout, job attitudes, and experiences for staff and travel nurses

	TI	JS	PS	EE	PF	WL	IT
Turnover intent	--	-.62∗	-.63∗	.47∗	.57∗	.35∗	.35∗
Job satisfaction	-.72∗	**.84**	.48∗	-.32∗	-.42∗†	-.26∗	-.10†
Pay satisfaction	-.49∗	.48∗	**.80**	-.27∗	-.40∗	-.38∗	-.37∗
Emotional exhaustion	.44∗	-.45∗	-.33∗	**.72**	.64∗	-.02†	.18
Physical fatigue	.55∗	-.60∗†	-.42∗	.73∗	**.92**	.36∗	.37∗
Workload	.41∗	-.42∗	-.32∗	.34∗†	.41∗	**.89**	.35∗
Illegitimate tasks	.48∗	-.45∗†	-.40∗	.32∗	.42∗	.50∗	**.81**

Note: Staff nurse correlations are below the main diagonal; travel nurse correlations are above the main diagonal; bolded values on main diagonal = coefficient alpha for combined sample. Measures included: Michigan Organizational Assessment Questionnaire (job satisfaction), Job Satisfaction Survey (pay satisfaction), Shirom-Melamed Burnout Measure (emotional and physical fatigue), Quantitative Workload Inventory (workload), and Matthews and colleagues' (2022) single-item measures (illegitimate tasks).^13^,^19^,^21^-^23^

∗ Correlation significantly different from zero at P < .05.

† Corresponding correlations between staff and travel nurses are significantly different from one another at *P* < .05.

## Discussion

A sample of licensed RNs in Florida received a survey designed to ascertain how travel nurses might differ from staff nurses in their experience of work. Specifically, the researchers wanted to determine if there were differences in burnout, job attitudes (overall job satisfaction, pay satisfaction, and turnover intentions), and perceptions of work assignments (illegitimate tasks and workload). There were several notable findings in terms of differences and similarities.

### Burnout

The survey included two dimensions of burnout: emotional exhaustion and physical fatigue. Results showed that travel nurses scored significantly higher on the physical fatigue component of burnout but not on emotional exhaustion. This pattern suggests that being a traveler might take a heavier physical toll, leaving the nurses physically worn out. Perceptions of workload didn't differ, so it's unlikely that the travelers were working harder but rather that the tasks they were assigned were more tiring or that demands outside of their work were contributing to fatigue.

Faller and colleagues, for example, discuss the challenges for travelers of adapting to a new location.^26^ Another possibility is that travelers have fewer close bonds inside or outside of work, which can lead to lower social support, a factor that has been linked to burnout in nurses.^27^,^28^ The lack of difference in emotional exhaustion suggests that being a traveler didn't take a greater emotional toll, so emotional distress isn't a likely explanation for physical fatigue.

### Job attitudes

There was no difference between travel and staff nurses in their overall job satisfaction or pay satisfaction. Overall satisfaction is driven by many factors including rewards (such as compensation), work colleagues (other nurses and supervisors), and the nature of work.^29^ The motivations and expectations of travelers are different from those of staff nurses, so the factors that are important to each group are likely different.^26^ This can result in the two types of nurses having the same level of overall job satisfaction for different reasons.

One facet for which there was no difference was pay satisfaction. Given the large disparity in pay between travel and staff nurses, one might expect that pay satisfaction would differ. On the contrary, the means were almost identical at approximately 11 on a scale ranging from 4 to 24. This suggests that the pay satisfaction for both groups is toward the dissatisfied end of the scale; in other words, they're equally dissatisfied. One likely explanation for similar levels of dissatisfaction is that travel nurses don't feel that the higher rate of pay is enough to compensate for the personal costs and inconvenience of being a traveler. Furthermore, travel nurses work through agencies that take a portion of the funds allotted for the traveler, and perhaps some nurses feel that their cut is unfair, thus contributing to pay dissatisfaction.

There was a disconnect between comparative results for turnover intentions and job satisfaction. The two variables were highly correlated (-.62 for travel nurses and -.72 for staff nurses), so one would expect that differences between the two types of nurses would be consistent across the two variables. However, travel nurses' turnover intentions were significantly higher, but the difference for job satisfaction wasn't statistically significant. Perhaps there were additional factors driving travel nurse turnover intentions other than job satisfaction.

For example, when on assignment, travel nurses experience a transient lifestyle, living away from family and in temporary quarters, often with limited social contacts outside of work. These factors might have contributed to greater turnover intentions as some travelers struggled to remain in their travel assignments and contemplated leaving early. Another possibility is that some of the travelers interpreted the question to mean that they were going to leave the hospital at the end of their assignment as opposed to attempting to renew the travel assignment or transitioning to a staff nurse position. In that case, the meaning of turnover intentions wouldn't be the same for the two types of nurses, precluding comparison.

### Perceptions of work assignments

Elevated scores for travel nurses on illegitimate tasks are consistent with Ronnie's findings that travel nurses noted they'd received unfair task assignments.^10^ Likely the travel nurses in the current study sample felt that they were given assignments that were beneath their skill level. This might occur because nurse managers don't have sufficient opportunities to become fully aware of travel nurses' knowledge and skills, and because travel nurses don't have as much time to learn local policies and procedures.^30^

For staff nurses, there was a significant correlation between illegitimate tasks and job satisfaction, which is consistent with prior research in other occupations and industries.^31^ For travelers, however, there was a nonsignificant and near-zero correlation between illegitimate tasks and job satisfaction that was significantly smaller than the corresponding staff nurse correlation. Although the travelers perceived receiving more illegitimate tasks, it didn't translate into lower job satisfaction. Perhaps it's because the travelers viewed this as a temporary situation or they understood that illegitimate tasks are to be expected when a nurse is new to the hospital. These findings suggest the need for additional research to better understand what might drive job satisfaction in travel nurses.

Despite differences in perceptions of illegitimate tasks, workloads were perceived to be similar for nurses in both groups, suggesting that nursing managers are being careful to balance workloads. Thus, the greater burnout among travel nurses isn't likely due to being overworked, at least when compared with staff nurses. Therefore, the two groups of nurses differed in their perceptions of what they're assigned to do but not how much they're asked to do.

## Limitations

Perhaps the biggest limitation of this study is the low response rate. Only 30% of those receiving the invitation email opened it, but there's no way to know how many of the 70% saw the email because the address they provided to the Department of Health when they were licensed might no longer be used, and many invitations likely wound up in spam folders. Of those who opened the email, only those who were eligible would have clicked the survey link because the inclusion criteria were clearly stated in the invitation, which would have disqualified a sizeable proportion of the sample. Nevertheless, even allowing that only a minority of eligible nurses saw the email, the rate was less than ideal, raising concerns about the representativeness of the sample. That said, there's research from the occupational stress literature suggesting that response rate has little effect on the relationships among variables.^32^

A second limitation is that the study relied on nurses' self-reports of task assignments and workload. Although the study assessed how nurses perceive their tasks, it's not clear to what extent that reflects objective aspects of the job or the actual tasks that nurse managers assign. Additionally, the study's cross-sectional design can't shed light on the extent to which assignments might lead to burnout and dissatisfaction, and it's certainly possible that those who feel dissatisfied and exhausted perceive their assignments differently than counterparts who are having a more positive experience of work.

Finally, the sample was taken from licensed nurses in one US state. It isn't clear how well results would generalize to nurses in other states or in other countries that might have different staffing and pay practices.

## Implications for nurse leaders

The results of this study underscore three areas of concern for nurse leaders when managing travel nurses; they're more likely than staff nurses to 1) be physically fatigued, 2) feel their task assignments are inappropriate, and 3) consider leaving their assignment prematurely. Their higher level of fatigue can be due to conditions both at and beyond work. At work, their perceptions of the tasks they're given differ from staff nurses, which could lead to greater stress and fatigue. Being temporarily away from home and their personal support network of family and friends can exacerbate this situation. This suggests a two-prong approach to addressing the needs of travel nurses.

First, the travelers likely need greater support from managers and peers. Not only are they away from their home support system, but their temporary status also makes it difficult to develop strong bonds at work. Extra efforts to provide support with continual check-ins and offers of assistance, as well as encouraging staff nurses on the unit to do the same, would go a long way to making the travelers feel welcome and less stressed.

Second, nurse managers should be mindful of how they utilize travelers to create a positive experience that supports their ability to provide good patient care. Managers should make an effort to learn about the travelers' knowledge and skills, so they know how best to utilize their talents. They should implement ways to validate travel nurse competencies, so their talents are better applied on their units.

Furthermore, nurse managers should be sensitive to travel nurses' perceptions of receiving leftover tasks that underutilize their capabilities. They should be transparent in explaining why assignments are given and allow travel nurses to provide input when possible. Even if the assignment can't be changed, the travel nurse would understand why they're being asked to perform certain tasks.

## Promoting a positive experience

This study is among the first to compare the work experiences of travel nurses with those of staff nurses. Although they don't differ in emotional exhaustion, job satisfaction, or workload, travelers experience greater physical fatigue, illegitimate tasks, and turnover intention, which can lead to quitting an assignment early. If nurse leaders give greater attention to the experiences of travel nurses, it might enable these nurses to have a more positive experience that can contribute to better job performance, as well as a better use of their talents during their assignments.

## INSTRUCTIONS Travel nurse work experiences: A comparison of staff and travel nurses' burnout and job attitudes

### TEST INSTRUCTIONS

- Read the article. The test for this nursing continuing professional development (NCPD) activity is to be taken online at www.NursingCenter.com/CE.
- You'll need to create an account (it's free!) and log in to access My Planner before taking online tests. Your planner will keep track of all your Lippincott Professional Development online NCPD activities for you.
- There's only one correct answer for each question. A passing score for this test is 8 correct answers. If you pass, you can print your certificate of earned contact hours and access the answer key. If you fail, you have the option of taking the test again at no additional cost.
- For questions, contact Lippincott Professional Development: 1-800-787-8985.
- Registration deadline is **June 5, 2026**.

### PROVIDER ACCREDITATION

Lippincott Professional Development will award 2.0 contact hours for this nursing continuing professional development activity.

Lippincott Professional Development is accredited as a provider of nursing continuing professional development by the American Nurses Credentialing Center's Commission on Accreditation.

This activity is also provider approved by the California Board of Registered Nursing, Provider Number CEP 11749 for 2.0 contact hours. Lippincott Professional Development is also an approved provider of continuing nursing education by the District of Columbia, Georgia, Florida, New Mexico, South Carolina, and West Virginia, CE Broker #50-1223. Your certificate is valid in all states.

Payment: The registration fee for this test is $21.95.
